# Characterization of the complete chloroplast genome of the rare medicinal plant: *Mandragora caulescens* (Solanaceae)

**DOI:** 10.1080/23802359.2024.2368213

**Published:** 2024-06-20

**Authors:** Heqin Ma, Erdong Zhang, Yajing An, Yuqing Wei, Lei Zhang

**Affiliations:** School of Biological Science & Engineering, Key Laboratory of Ecological Protection of Agro-pastoral Ecotones in the Yellow River Basin National Ethnic Affairs Commission of the People’s Republic of China, North Minzu University, Yinchuan, PR China

**Keywords:** *Mandragora caulescens*, Solanaceae, chloroplast genome, phylogenetic analysis

## Abstract

In this study, we assembled high-quality chloroplast genomes of *Mandragora caulescens* through a reference-guided approach using high-throughput Illumina sequencing reads. The resulting chloroplast genome assembly displayed a typical quadripartite structural organization, comprising a large single-copy (LSC) region of 85,233 bp, two inverted repeat (IR) regions of 25,685 bp each, and a small single-copy (SSC) region of 18,207 bp. The chloroplast genome harbored 141 complete genes, and its overall GC content was 38.0%. In maximum-likelihood (ML) and Bayesian inference (BI) trees, the 19 Solanaceae species formed a monophyletic group, dividing into two main clades. *M. caulescens* and *Nicandra physalodes* formed a monophyletic group, suggesting a close relationship between the two species. The *M. caulescens* cp genome presented in this study lays a good foundation for further genetic and genomic studies of the Solanaceae.

## Introduction

*Mandragora caulescens* C. B. Clarke (Clarke 1883), a rare medicinal plant, belongs to the tribe Solaneae (Solanaceae). It mainly grows in the meadows and alpine meadows (2200–4200 m a.s.l.) in western Sichuan, northwestern Yunnan, and eastern Tibet of China (Zhang et al. 1994; Mabberley 2017). The roots of this plant contain high levels of total alkaloids, with the main components being anisodamine and apoatropine (Wang et al. 2002; Wan et al. 2011). Previous research has indicated that both the roots and aerial parts of the plant are commonly used in traditional Chinese medicine, renowned for their ability to alleviate pain and treat certain skin conditions (Maity et al. 2019).

The chloroplast is a semi-autonomous eukaryotic organelle that has a small genome (cp genome) that interacts with the nuclear and mitochondrion genome to provide the biochemical machinery for energy conversion (Li et al. 2015; Hollingsworth et al. 2016). Recent utilization of the complete chloroplast genome in addressing phylogenetic issues has been increasing, attributed to its distinct characteristics and its ability to reveal valuable information worth considering (Guo et al. 2017; Zhang et al. 2018; Xie et al. 2022; Ran et al. 2024). The complete chloroplast genomes have been extensively employed in resolving some lingering queries in plant taxonomy recently (Hu et al. 2016; Yang et al. 2022). Besides the evolutionary aspect, the chloroplast genome holds significant implications in chloroplast transformation (Daniell et al. 2008). A comprehensive examination of *Mandragora caulescens* could have notable implications for comprehending the origin and evolution of the Solanaceae. Previous phylogenetic investigations of *M. caulescens* primarily relied on a limited number of chloroplast genes or ITS (Volis et al. 2015, 2018), Nevertheless, the chloroplast genome of *Mandragora caulescens* remains unreported.

In this study, we assembled and analyzed the complete chloroplast genome of *M. caulescens* for the first time.

## Materials

**Figure 1.**
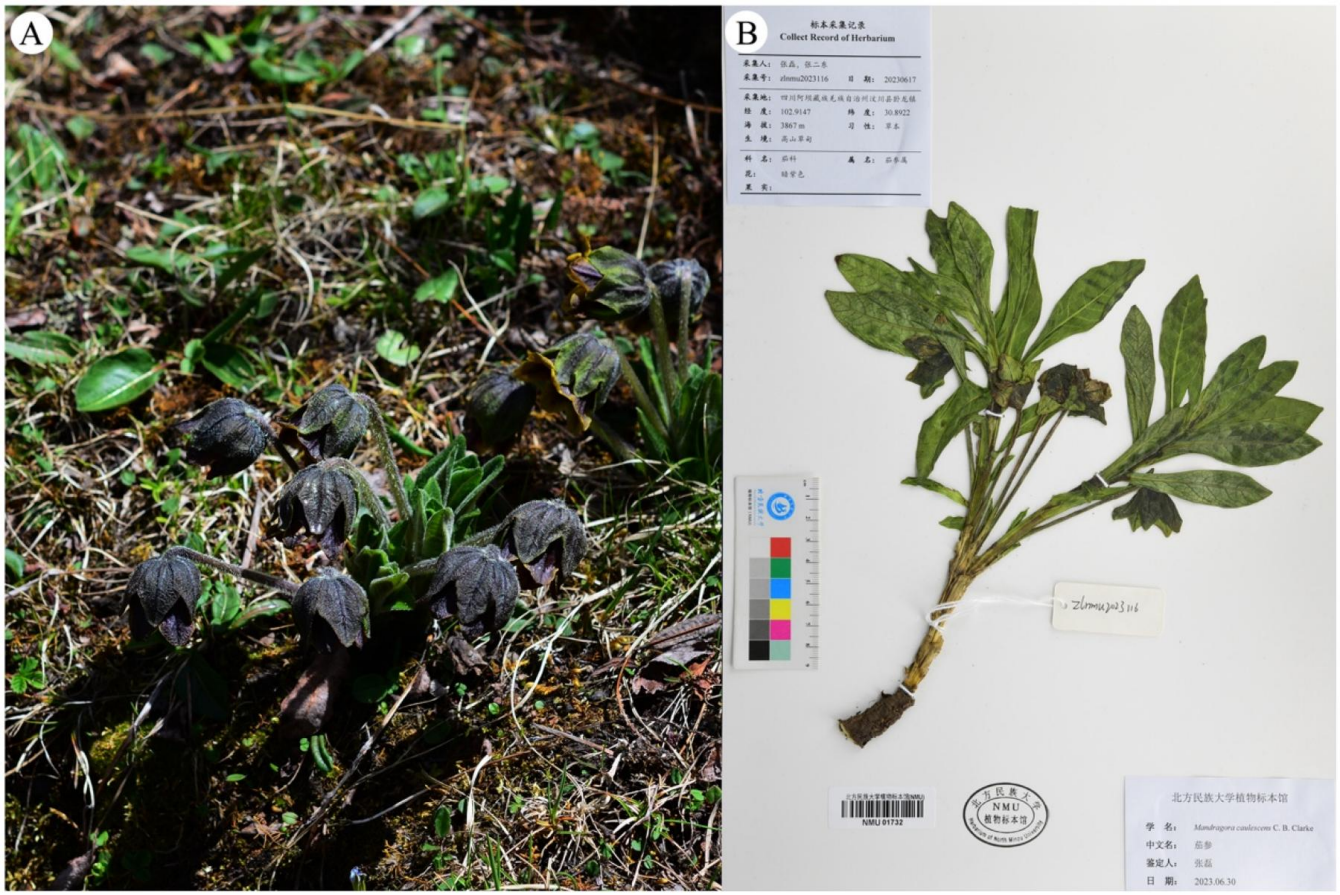
(A) An individual plant of *M. caulescens* taken from Balang mountains, Wenchuan, Sichuan, China (photoed by Dr. Lei Zhang). (B) Herbarium of *M. caulescens* (Calyx campanulate, divided to halfway; Corolla dark purple).

Fresh leaves of florescence of *M. caulescens* were gathered from Balang mountains (Wenchuan, Sichuan, China; coordinates: 102.9147 E, 30.8922 N) (by Lei Zhang: zhangsanshi-0319@163.com) (Figure 1(A)), and desiccated using silica gel. The voucher specimen was archived in the Herbarium of North Minzu University with an accession number of zlnmu2023116 (Figure 1(B)). 0.5 g of the dry leaf sample was utilized for DNA extraction.

## Methods

Total genomic DNA was isolated with a modified CTAB method (Doyle and Doyle 1987). The NEBNext DNA Library Kit was employed to create the sequencing libraries following the manufacturer’s instructions. DNA was randomly sheared to a size of 350 bp. This library was sequenced on the Illumina NovaSeq 6000 platform with 150 bp paired-end read length. We acquired 6.2 Gb of high-quality paired-end reads for *M. caulescens*. After eliminating the adapters, two approaches were used to assemble the cp genome, we *de novo* assembled the cp genome *M. caulescens* using NOVOPlasty 4.1 (Dierckxsens et al. 2017) with the specified parameters: k-mer = 39 and genome range 120,000–200,000 bp. The other approach was assembled using GetOrganelle (Jin et al. 2020). Sequence lengths obtained by the two methods are similar. The complete chloroplast genome sequence of *Physalis angulata* (MH045574) was utilized as a reference. Plann v1.1 (Huang and Cronk 2015) was employed for annotating the chloroplast genome, and Geneious v11.0.3 (Kearse et al. 2012) was used for correcting the annotation. The sequencing depth coverage was conducted by Samtools (Li et al. 2009). To further elucidate the phylogenetic placement of *M. caulescens* in Solanaceae, the chloroplast genomes of 19 representative species were retrieved from NCBI GenBank to reconstruct the chloroplast genome phylogenetic tree, with *Convolvulus arvensis* serving as an outgroup. Protein-coding genes (PCGs) were extracted from the GenBank formatted file containing 20 plastomes using customized Perl scripts, removing start and end codons. A total of 56 PCGs were retained for all species. Each PCG was aligned using PRANK v.130410 (Löytynoja and Goldman 2008) according to the translated amino acid sequences. Phylogenetic analyses were performed for each dataset (CDS and WP) using both maximum-likelihood (ML) and Bayesian inference (BI) strategies. We used RAxML v8.1.24 (Stamatakis 2014) to conduct ML analyses with the GTR + Γ model. The optimal model (GTR + I + G) was identified using jModeltest and BI analysis was conducted in MrBayes v 3.2.6 (Ronquist et al. 2012). FigTree v1.4.2 (Rambaut 2012) was subsequently utilized to visualize the phylogeny. The results of the comparative analysis of the CPGs were visualized with the mVISTA program (Frazer et al. 2004) and the annotated CPG of *Capsicum annuum* was used as the reference in the LAGAN mode (Brudno et al. 2003).

## Results

**Figure 2.**
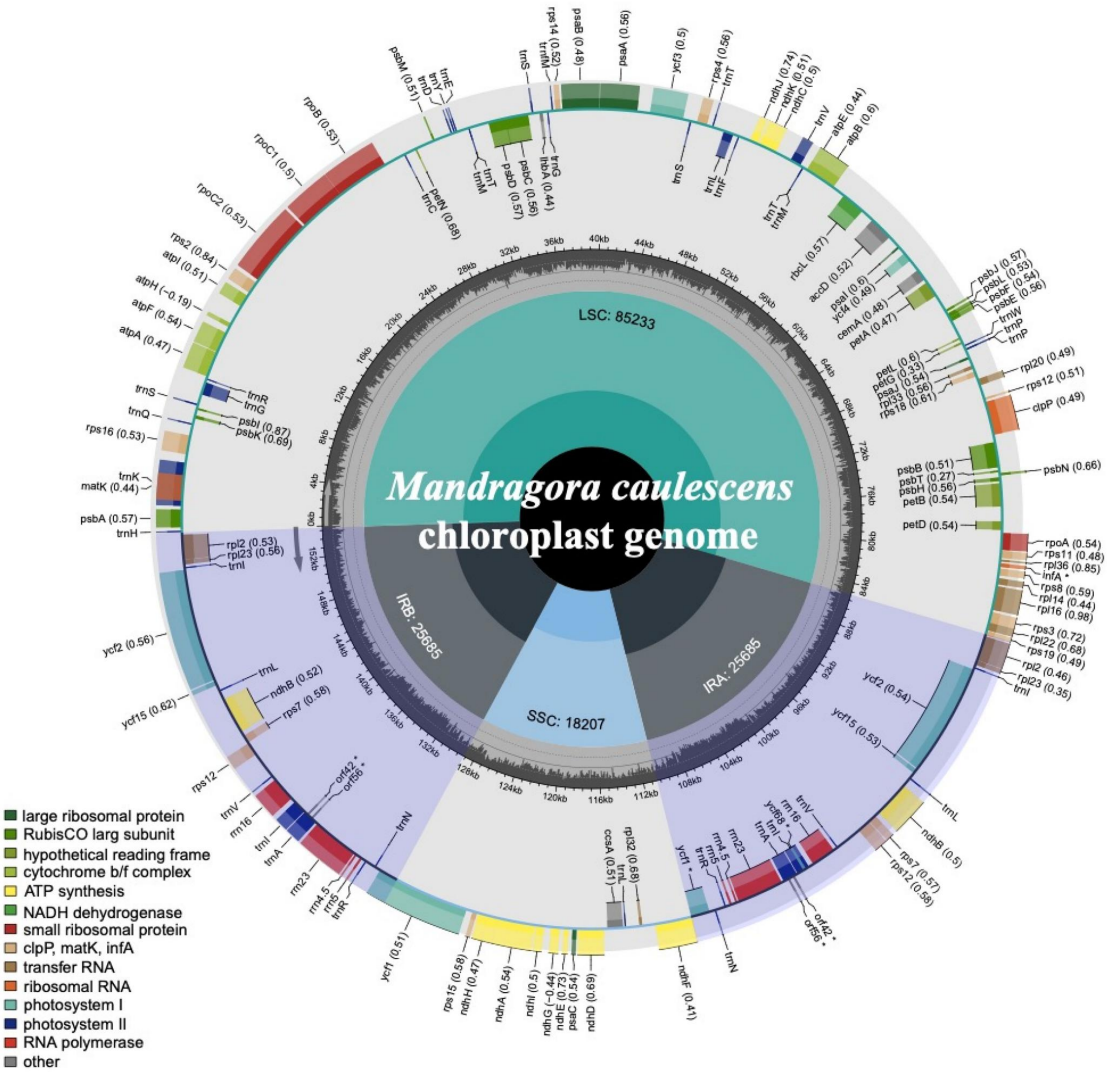
The detailed genome map of *M. caulescens* cp genome. The large single-copy (LSC), small single-copy (SSC) region and two inverted repeat regions (IRA and IRB), and GC content (light gray) are shown in the inside track. Gene models including protein-coding genes, tRNA genes and rRNA genes are shown with various colored boxes in the outer track.

**Figure 3.**
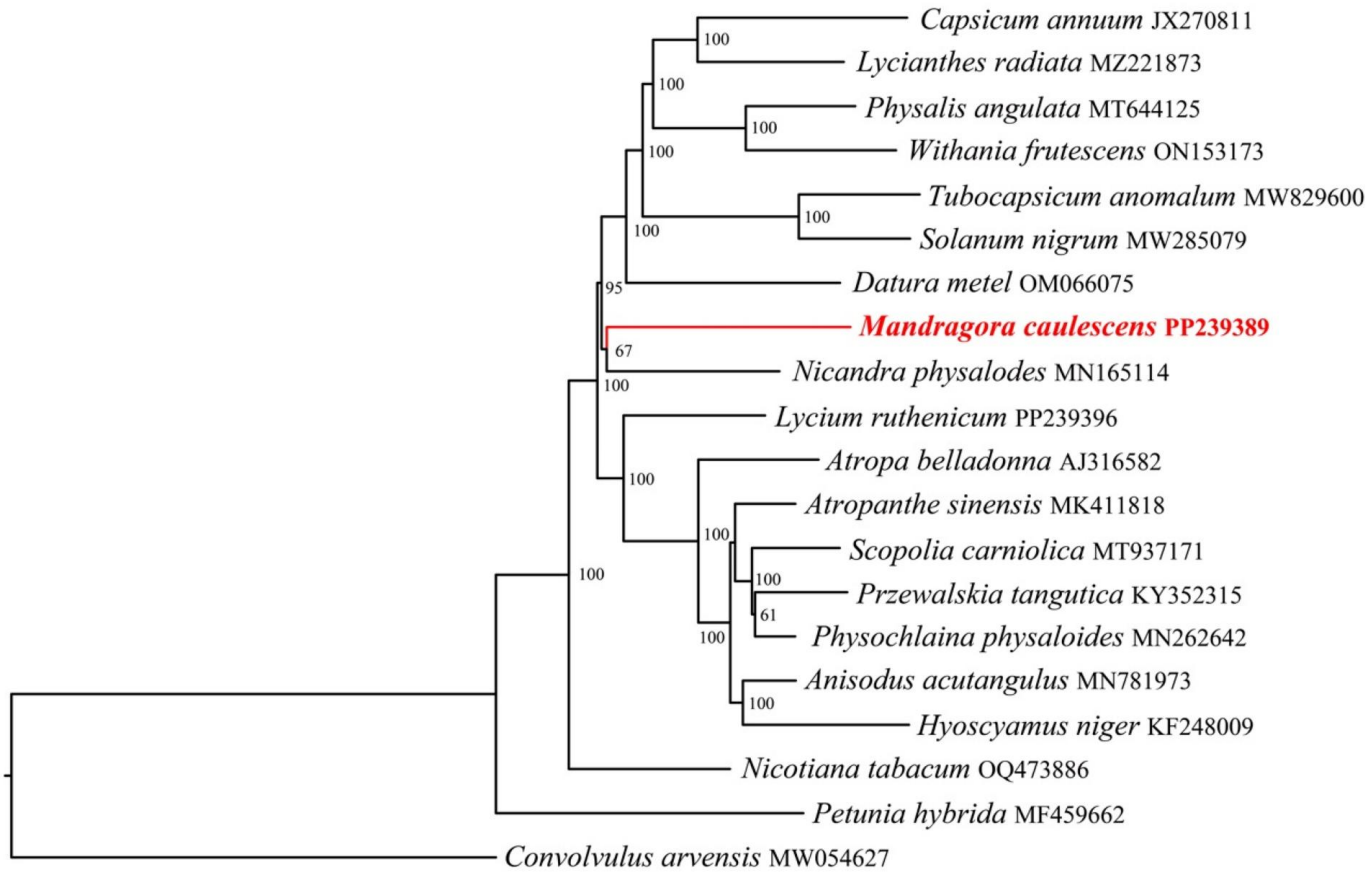
Phylogenetic tree obtained using the maximum-likelihood (ML) methods of Solanaceae species based on 56 PCGs. GenBank accession numbers: *Anisodus acutangulus* MN781973 (Tian et al. 2020), *Atropa belladonna* AJ316582 (Sahoo and Rakshit 2022), *Atropanthe sinensis* MK411818 (Jiang et al. 2019), *Capsicum annuum* JX270811 (Raveendar et al. 2017), *Convolvulus arvensis* MW054627 (Wang et al. 2021), *Datura metel* OM066075 (Ngai et al. 2022), *Hyoscyamus Niger* KF248009 (Sanchez-Puerta and Abbona 2014), *Lycianthes radiata* (MZ221873), *Lycium ruthenicum* (PP239396), *Mandragora caulescens* (PP239389), *Nicandra physalodes* MN165114 (Chen and Zhang 2019), *Nicotiana tabacum* (OQ473886), *Petunia hybrida* MF459662 (Wong et al. 2019), *Physalis angulate* MT644125 (Sun et al. 2021), *Physochlaina physaloides* MN262642 (Tong et al. 2019), *Przewalskia tangutica* KY352315 (Zhang and Chi 2019), *Scopolia carniolica* MT937171 (Gandini et al. 2021), *Solanum nigrum* (MW285079), *Tubocapsicum anomalum* MW829600 (Wang et al. 2021), and *Withania frutescens* ON153173 (Ramadan et al. 2023).

After quality control and preprocessing, we obtained at least four gigabases (Gb) of whole-genome sequencing data. The clean reads were used to assemble high-quality chloroplast genomes through a reference-guided approach. The total chloroplast genome of *M. caulescens* (PP239389) was 154,810 bp long, with average depths of 7027×, 4748.07×, and 354× for maximal, minimal, and average, respectively (Supplementary Fig. S1). It exhibited a typical quadripartite structural organization, comprising a large single-copy (LSC) region of 85,233 bp, two inverted repeat (IR) regions of 25,685 bp each, and a small single-copy (SSC) region of 18,207 bp (Figure 2, Supplementary Fig. S2). The chloroplast genome harbored 141 complete genes (Supplementary Table S1), including 86 PCGs, eight ribosomal RNA genes (rRNAs), and 40 tRNA genes (40 tRNAs). Most genes were present in a single copy, while 23 genes were duplicated, encompassing four rRNAs (rrn16, rrn23, rrn4.5, and rrn5), nine tRNAs (trnA-UGC, trnI-CAU, trnI-GAU, trnL-CAA, trnM-CAU, trnN-GUU, trnR-ACG, trnT-GGU, and trnV-GAC), and 10 PCGs (ndhB, rpl2, rpl23, rps7, rps12, ycf1, ycf2, ycf15, orf42, and orf56), with ycf1, orf42, and orf56 being pseudogenes. Additionally, the chloroplast genome contained 1 trans-splicing gene (Supplementary Fig. S3) and 12 cis-splicing genes (Supplementary Fig. S4). The overall GC content of the chloroplast DNA was 38.0%, with the LSC, SSC, and IR regions having corresponding values of 36.1%, 32.3%, and 43.1%, respectively. Both ML and BI trees confirmed the placement of *M. caulescens* within the Solanaceae family (Figure 3, Supplementary Fig. S5). In these trees, the 19 Solanaceae species formed a monophyletic group, dividing into two main clades with strong support (BS, PP = 100%, 1) (Figure 3, Supplementary Fig. S5). *M. caulescens* and *Nicandra physalodes* formed a monophyletic group (BS, PP = 85%, 1), suggesting a close relationship between the two species.

## Discussion and conclusions

Previous research has indicated that land plant cp genomes typically range from 120 to 160 kb (Wicke et al. 2011). In this investigation, in this study, the complete cp genome of *M. caulescens* was assembled for the first time, and the structure of this species was annotated. It exhibited a standard quadripartite structure with a total sequence length of 154,810 bp, encompassing the LSC region (85,233 bp), the SSC region (18,207 bp), and two identical IR regions (25,685 bp). The GC content of *M. caulescens* plants is 38.0%, consistent with findings from various angiosperm studies where the highest GC content was observed in the IR regions (Wu et al. 2020).

The cp genome serves as a focal point in molecular biology research and has emerged as a current area of interest for species genealogy identification (Ran et al. 2024). In this study, a phylogenetic tree was developed utilizing the BI method and the ML method. The tree revealed that the cp the cp genomes of Solanaceae species clustered together with strong support, with *M. caulescens* and *Nicandra physalodes* forming a monophyletic group. This phylogenetic result was consistent with Volis et al. (2018). The alignments indicated high sequence similarity among the CPGs of the 19 Solanaceae species (Supplementary Fig. S6). However, sequence divergence in non-coding regions was greater than that in coding regions. Further investigation of *M. caulescens* is necessary, including additional studies at the population level and genome analysis, as well as examining the distribution of the population. Only through thorough analysis can the adaptive differentiation of *M. caulescens* be understood. In addition, expanding the collection of Solanaceae cp genomes will provide deeper insights into the evolution of this ecologically and economically important plant family.

## Supplementary Material

Supplemental Material

## Data Availability

The sequenced data supporting the findings of this study are openly available in NCBI (https://www.ncbi.nlm.nih.gov/) under the accession no. PP239389. The associated BioProject, SRA, and Bio-Sample numbers are PRJNA1081155, SRR28114605, and SAMN40149505, respectively.
