# Supplementary material for: Characterization of the complete chloroplast genome of the rare medicinal plant: Mandragora caulescens (Solanaceae)
Source: Mitochondrial DNA B Resour. 2024 Jun 20;9(6):812–7. doi: 10.1080/23802359.2024.2368213 (PMC11191837; doi:10.1080/23802359.2024.2368213)
Supplement: Supplemental Material [file TMDN_A_2368213_SM5927.docx]

Table S1 Genes present in the *M. caulescens* cp genome

|  | Group of genes | Name of genes |
| --- | --- | --- |
| 1 | ATP synthase | *atp*A, B, E, F^b^, H, I |
| 2 | NADH dehydrogenase | *ndh*A^b^, B^b,c^, C, D, E, F, G, H, I, J, K |
| 3 | Cytochrome b/f | *pet*A, B^b^, D, G, L, N |
| 4 | Photosystem I | *psa*A, B, C, I, J |
| 5 | Photosystem II | *psb*A, B, C, D, E, F, H, I, J, K, L, M, N, T |
| 6 | Large subunit of ribosome | *rpl*14, 16^b^, 2^b,c^, 20, 22, 23^c^, 32, 33, 36 |
| 7 | Small subunit of ribosome | *rps*2, 3, 4, 7^c^, 8, 11, 12^a,c,d^, 14, 15, 16^b^, 18, 19 |
| 8 | DNA dependent RNA polymerase | *rpo*A, B, C1^b^, C2 |
| 9 | Subunit of rubisco | *rbc*L |
| 10 | Conserved open reading frames | *ycf*1^c^, 2^c^, 3^a^, 4, 15^c^ |
| 11 | Ribosomal RNAs | rrn4.5S^c^, 5S^c^, 16S^c^, 23S^c^ |
| 12 | Pseudogene | *ycf*68, *inf*A, *orf*42^c^, *orf*56^c^ |
| 13 | Transfer RNAs | *trn*A(UGC)^c^, C(GCA), D(GUC), E(UUC), F(GAA), fM(CAU), G(GCC), G(UCC), H(GUG), I(GAU)^c^, I(CAU)^c^, K(UUU), L(UAA), L(CAA)^c^, L(UAG), M(CAU)^c^, N(GUU)^c^, P(UGG), P(GGG), Q(UUG), R(ACG)^c^, R(UCU), S(GCU), S(GGA), S(UGA), T(UGU), T(GGU)^c^, V(UAC), V(GAC)^c^, W(CCA), Y(GUA) |
| 14 | Other proteins | *lhb*A, *cem*A, *clp*P^a^, *mat*K, *ccs*A, accD |

^a^ Gene containing two introns

^b^ Gene containing a single intron

^c^ Two gene copies in IRs

^d^ Gene divided into two independent transcription units


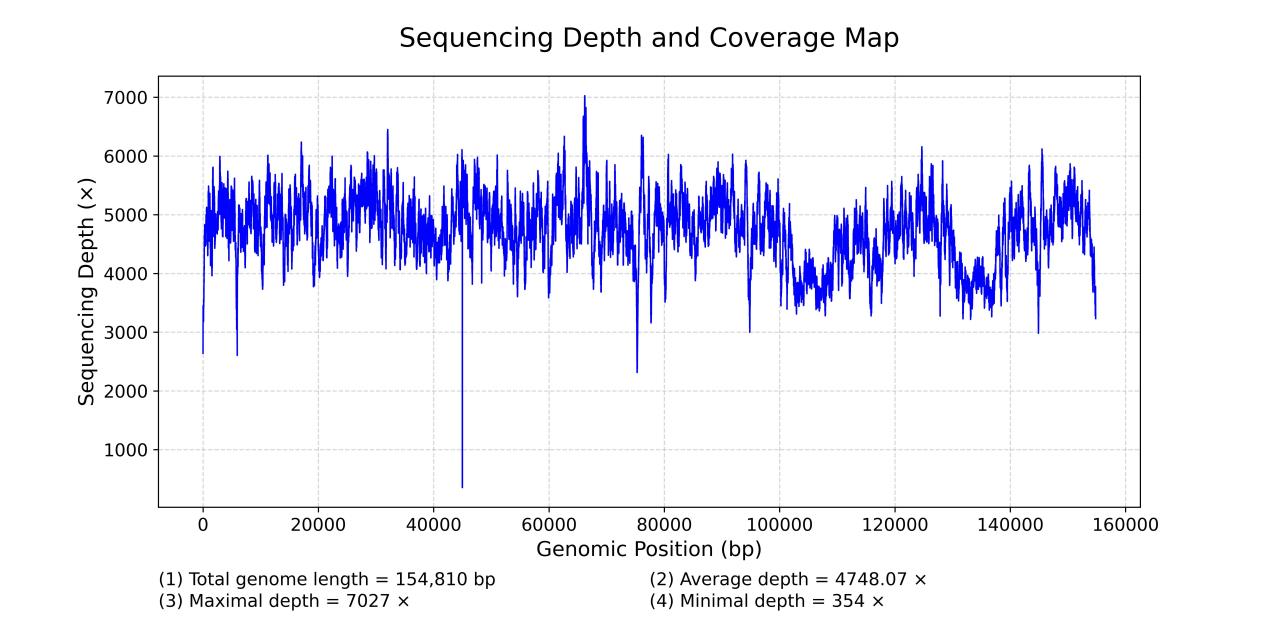


**Figure S1** Coverage depth distribution of the *M. caulescens*.


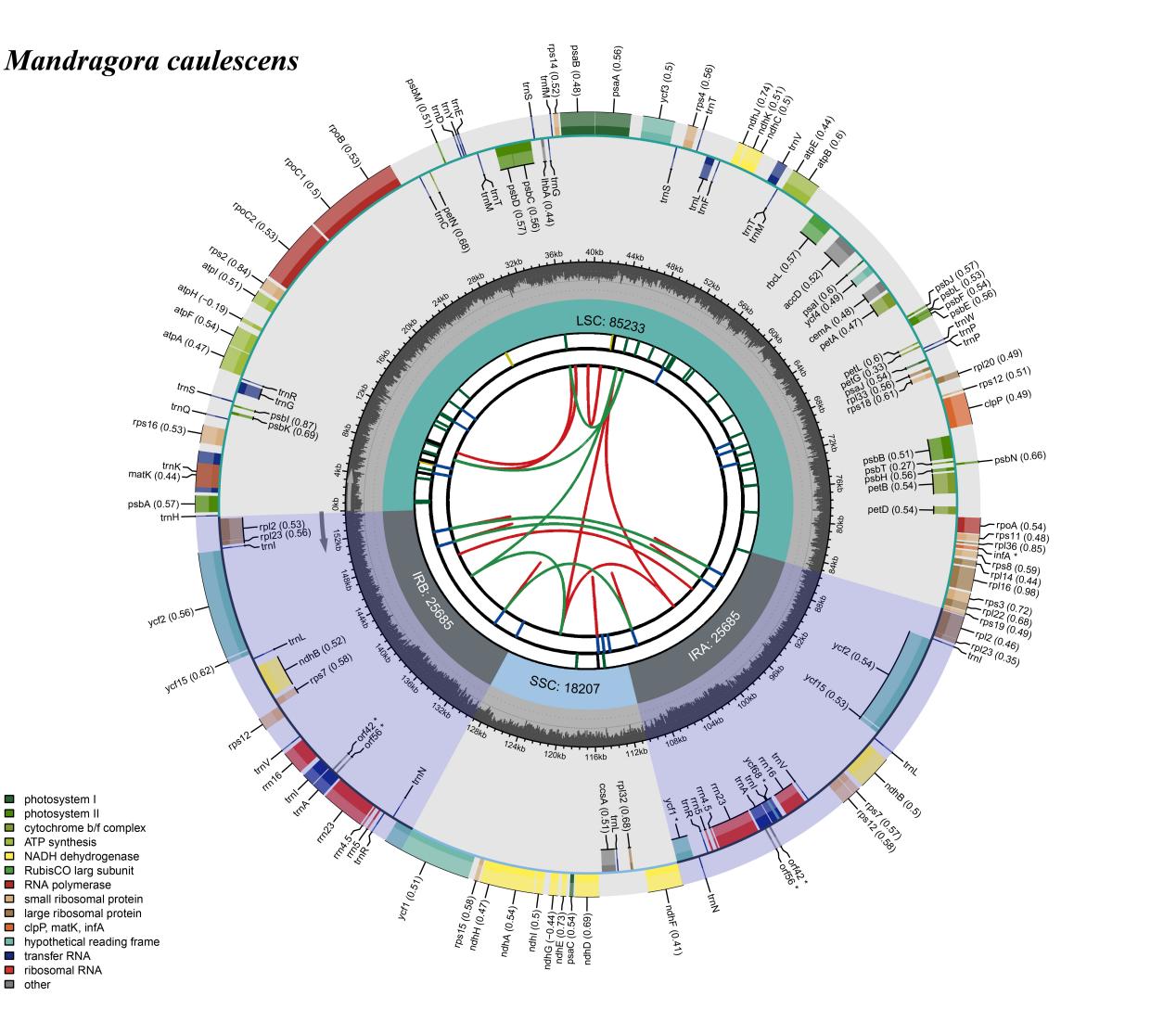


**Figure S2** Collinearity map of *M. caulescens*.


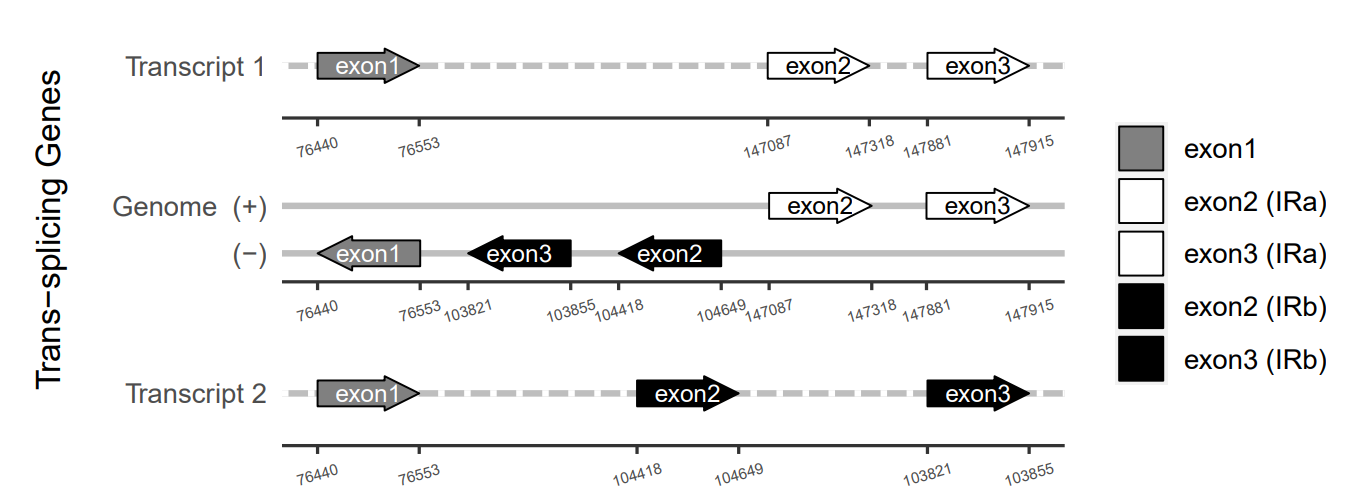


**Figure S3** Structure of trans-splicing genes in the *M. caulescens*.


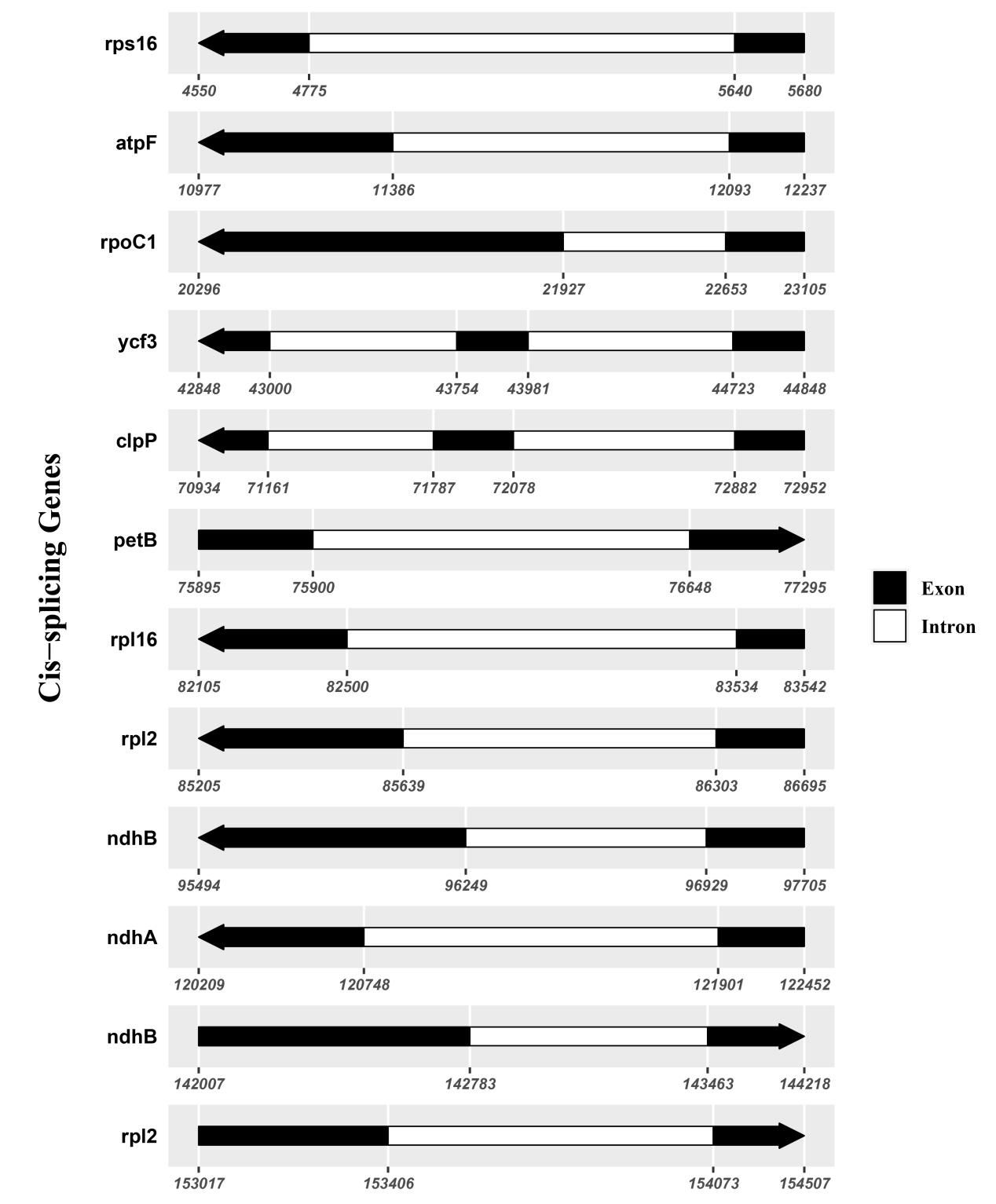


**Figure S4** Structure of Cis-splicing genes in the *M. caulescens*.


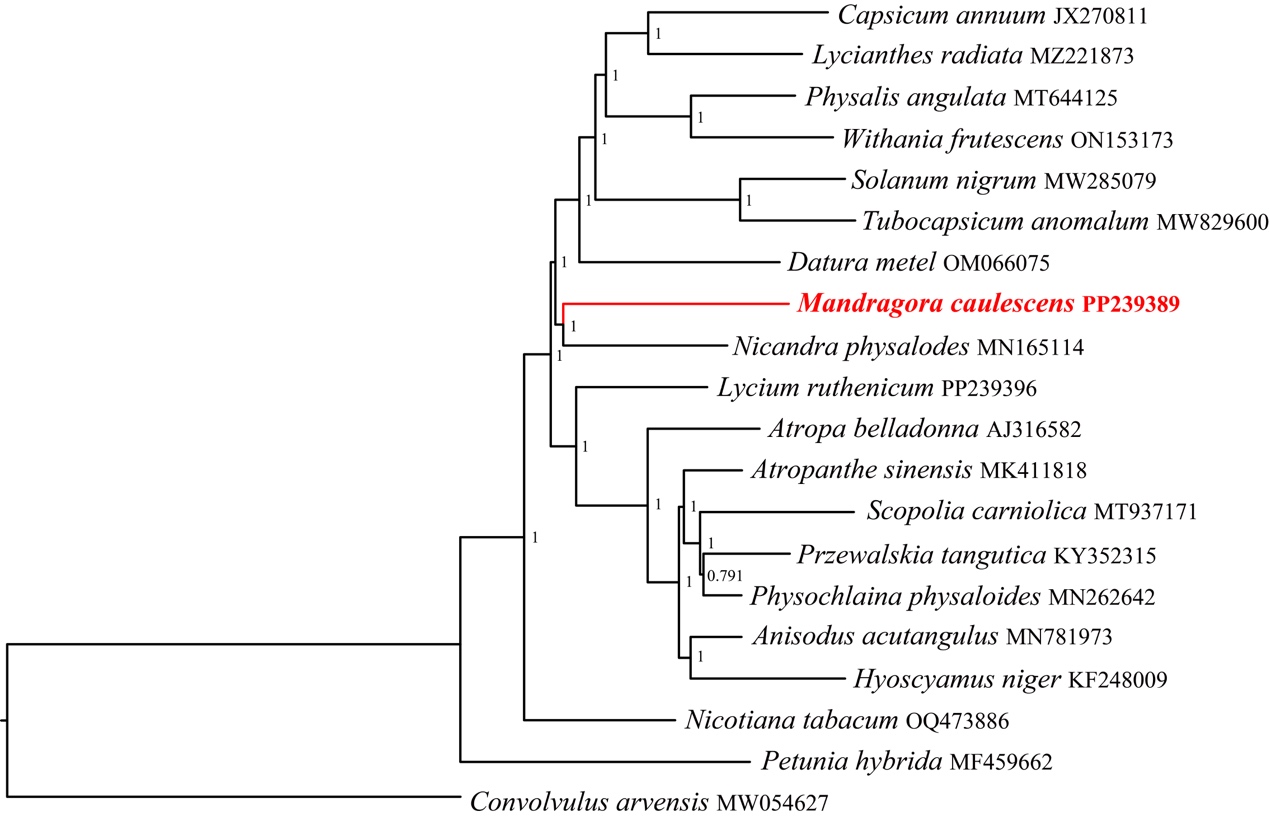


**Figure S5** Phylogenetic tree obtained using the Bayesian inference (BI) methods for the Solanaceae family based on 77 PCGs.


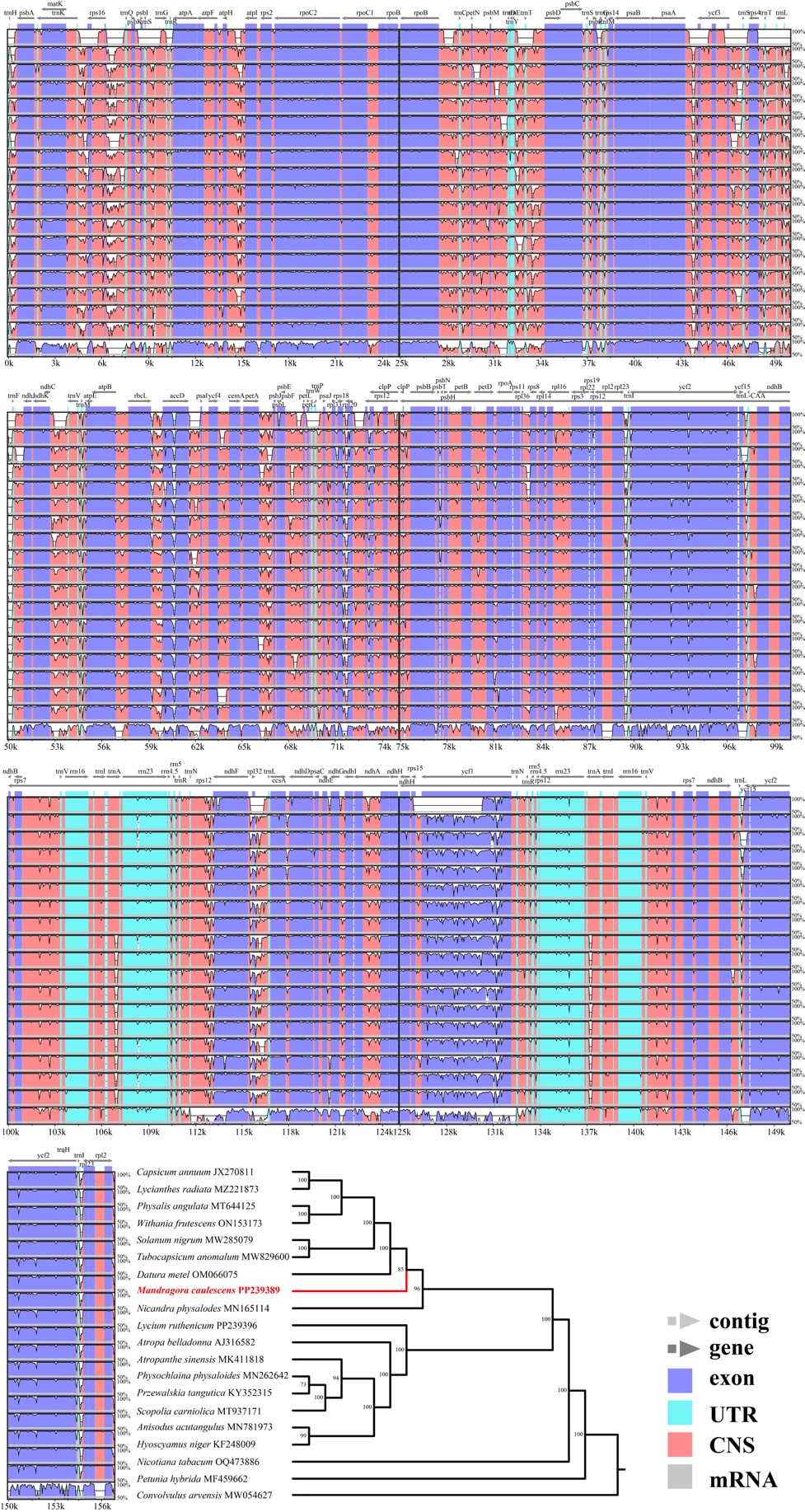


**Figure S6** Sequence alignment of the CPGs of 19 Solanaceae species. The alignment was performed using the mVISTA program and the Capsicum annuum chloroplast genome was used as a reference. The y-axis indicated the degree of identity ranging from 50 to 100%. Coding and non-coding regions were marked in blue and red, respectively. Black arrows indicated the position and direction of each gene. CNS: conserved non-coding sequences
